# Cartilage loss is greater in knees with virtual joint replacement status than in matched controls without

**DOI:** 10.1016/j.ocarto.2025.100658

**Published:** 2025-08-05

**Authors:** Felix Eckstein, Wolfgang Wirth, Ali Guermazi, Frank Roemer, Michael Nevitt, Christoph Ladel, Leena Sharma, David J. Hunter, C. Kent Kwoh

**Affiliations:** a Research Program for Musculoskeletal Imaging, Center for Anatomy and Cell Biology, and Ludwig Boltzmann Institute of Arthritis and Rehabilitation (LBIAR), Paracelsus Medical University (PMU) Salzburg, Austria; b Chondrometrics GmbH, Freilassing, Germany; c Department of Radiology, Chobanian & Avedisian Boston University School of Medicine, Boston, MA, USA; d Department of Radiology, Universitätsklinikum Erlangen & Friedrich-Alexander-Universität Erlangen-Nürnberg (FAU), Erlangen, Germany; e Department of Epidemiology and Biostatistics, University of California, San Francisco, CA, USA; f CHL4special Consultancy, Darmstadt, Germany; g Department of Medicine, Feinberg School of Medicine, Northwestern University, Chicago IL, USA; h Rheumatology Department, Royal North Shore Hospital. & Sydney Musculoskeletal Health, Kolling Institute, University Sydney, Sydney, Australia; i Department of Epidemiology, Grad. Sch. of Public Health, University of Pittsburgh, Pittsburgh, PA, USA; j Division of Rheumatology and Clinical Immunology, University of Pittsburgh School of Medicine, Pittsburgh, PA, USA; k Division of Rheumatology and University of Arizona Arthritis Center, University of Arizona College of Medicine, Tucson, AZ, USA

**Keywords:** Knee osteoarthritis, Knee replacement, Virtual, Cartilage loss, MRI

## Abstract

**Objective:**

We examined whether the trajectory of femorotibial cartilage loss differs between knees meeting a clinically defined virtual knee replacement (vKR) status based on patient-reported outcomes vs. those with low probability.

**Design:**

vKR cases (highest 10 ​% of probabilities for having surgical KR) were selected using knee pain and quality of life criteria, developed from knees with actual KR. Knees reaching such symptom state at 48 months (M) follow-up (vKR case 60 ​M) were matched 1:1 with vKR controls (lowest 20 ​% probability) by sex, age, and baseline radiographic status. Of 65 knees displaying vKR case status at 60 ​M; 33 maintained or increased pain and QOL levels at 72 ​M (vKR+/+); 32 did not (vKR+/−). The thickness of medial and lateral tibial and femoral cartilages was determined from MRI, at up to five annual visits prior to 60 ​M.

**Results:**

vKR case knees displayed substantially greater central medial femorotibial compartment cartilage thickness loss 2 years prior to reaching case status (−151 ​± ​337 [mean ​± ​SD] vs. −38 ​± ​249 ​μm; odds ratio [OR] 1.95 (95 ​% confidence interval: 1.23–3.08). Cartilage loss did not apparently differ between vKR+/+ and vKR +/− knees (p ​= ​0.73).

**Conclusions:**

Substantially greater cartilage thickness loss was detected in knees reaching vKR case status defined by patient-reported clinical criteria vs. vKR controls. This was found independently of whether this status was maintained at a later annual visit. The observed association suggests greater knee cartilage loss to be prospectively related to worse clinical outcome. It indicates further that the vKR criterion used here may be useful to explore relationships with other structural (imaging) assessments.

## 1 Introduction

Symptomatic therapy for knee osteoarthritis (OA) commonly demonstrates only modest effect sizes and numerous undesirable side effects; therefore knee replacement (KR) often remains the last resort for patients with progressive knee osteoarthritis (OA) [1,2]. The absence of disease modifying OA drug (DMOAD) therapy, which changes the natural progression of the disease and addresses both symptoms and structural pathology, currently represents an urgent unmet medical need [3]. For DMOAD trials, surgical KR is considered a valid clinical endpoint. However, estimates of required sample sizes range from 3.000 to 18.000 participants over 3 years [3,4], numbers that are prohibitive in terms of resources and feasibility.

Cartilage loss was found significantly greater prior to surgical KR, when compared with non-replaced control knees [[5], [6], [7], [8], [9], [10], [11]], even when case and control knees were matched for baseline radiographic disease severity (Kellgren Lawrence grade ​= ​KLG), sex, and age [[6], [7], [8]]. However, clinical indications for performing KR, or not, are highly variable and depend on factors not objectively related to clinical disease status [12,13]. Decision making for KR is thus strongly influenced by the patient's expectation of functional capability after surgery, socio-economic and insurance status, willingness to undergo surgery, comorbidities, structural disease status, and other factors [[14], [15], [16]]. To overcome these limitations, virtual KR definitions have been developed based on outcome measures, independent of whether someone has actually received surgical KR or not [13,[17], [18], [19]]. Such definitions can potentially serve as “more objective” and robust outcome in clinical trials than surgical KR alone, involving smaller sample sizes. In contrast to some of the previous definitions, the one used here is based solely on patient-reported outcomes (PROs), but not on imaging/radiography or other study endpoints. The use of multi-component endpoints, defined by surgical KR in combination with conservative thresholds of PROs of pain and function, has been reported to reduce the estimated sample size for DMOAD trials vs. surgical KR alone by 30–40 ​% [3]. However, whether virtual knee replacement (vKR) case status is also associated with faster rates of prior structural disease progression (e.g., quantitative cartilage loss) than vKR control status, has not been explored.

Investigating this relationship serves two purposes: a) to determine whether cartilage loss is prospectively related to PROs, and b) to determine whether the vKR definition exhibits similar relationships with structural disease progression as does surgical KR [[6], [7], [8]]. The specific objective therefore was to examine whether the trajectory of femorotibial cartilage loss differs between knees that attain vKR case status vs. their matched vKR controls with low probability of becoming a surgical KR case. Cartilage thickness change was determined by MRI, an imaging biomarker commonly used in efficacy trials of structural disease modification [[20], [21], [22], [23], [24], [25]].

## 2 Method

### 2.1 Study design

This study was ancillary to the Osteoarthritis Initiative (OAI) multi-center longitudinal cohort study (https://nda.nih.gov/oai) [26,27]. Participants were studied annually at four centers, with 3T MRIs acquired at baseline, 12, 24, 36, and 48 month (M) follow-up [26,27], and vKR status determined at 60 ​M. OAI participants were 45–79 years old, exhibited symptomatic KOA, or were at risk of symptomatic KOA in at least one knee. This study was approved by the Institutional Review Boards of the University of California and each of the Clinical Centers. (No 10–00532) [26,27].

**Fig. 1:**
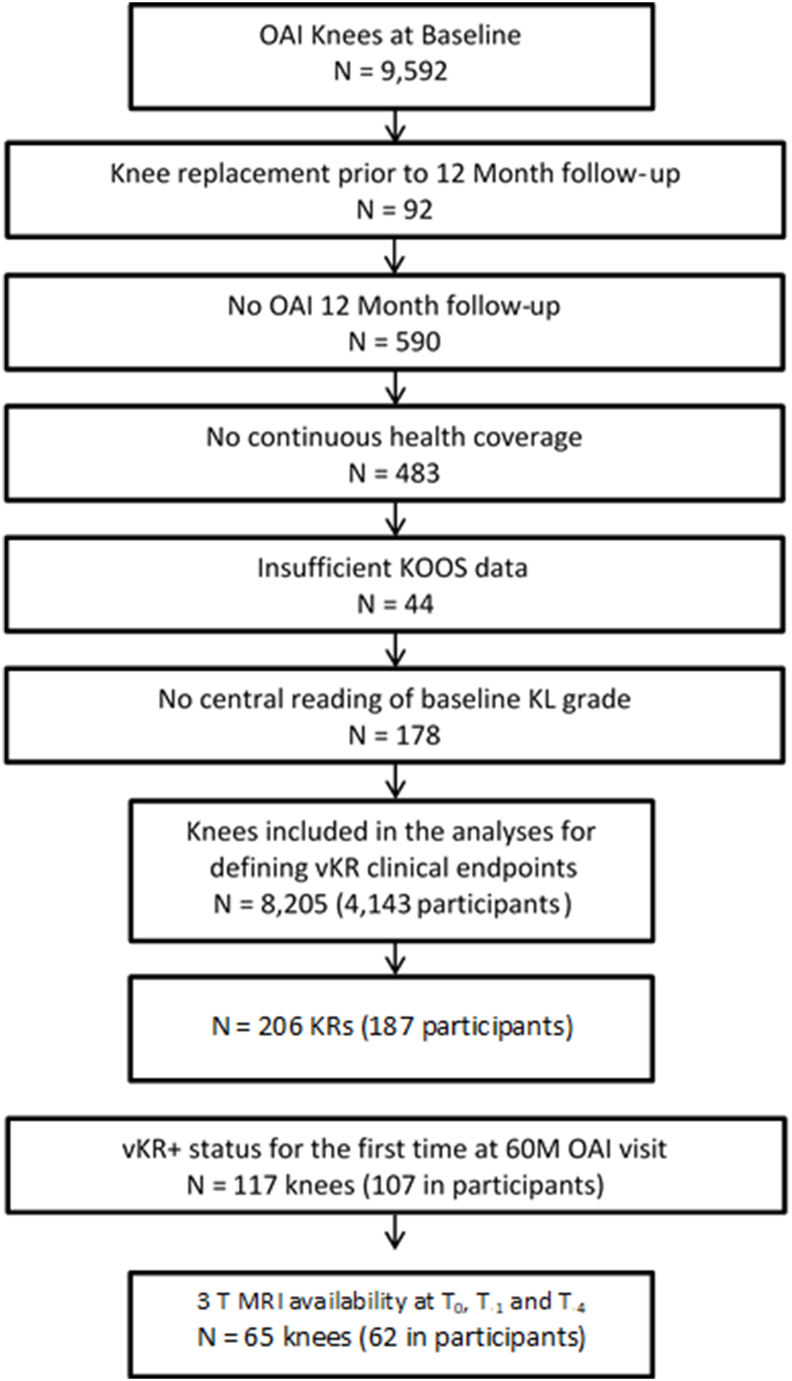
Flowchart of the inclusions and exclusions for the selection of OAI participant knees used in this analysis on the relationship of vKR case status with prior cartilage thickness loss. Of a total of 9592 OAI knees, those (n ​= ​92) with KR prior to 12 months (M) follow-up, lack of 12 ​M follow up (n ​= ​590), lack of continuous health coverage (n ​= ​483), insufficient KOOS data (n ​= ​44), and lack of central baseline radiograpic KL grade readings (n ​= ​178) were removed. Of the remaining 8205 knees, 206 had a surgical KR. In 117, vKR case status was first observed at 60 ​M, and of those 65 knees (in 62 participants) had cartilage MRI available from baseline through 48 ​M and a matched vKR control.

Of 9592 OAI baseline knees, 8205 were available to select vKR cases after eliminating those who lacked relevant data, had no follow up data or had a KR at 12 ​M, and did not have health care coverage (Fig. 1). After excluding prior KRs, 117 knees reached vKR case status for the first time at 60 ​M (see below), and 65 knee (of 62 participants) had (cartilage) MRI data availed from BL through 48 ​M (Fig. 1), and could be successfully matched with a vKR control (see below).

**Fig. 2:**
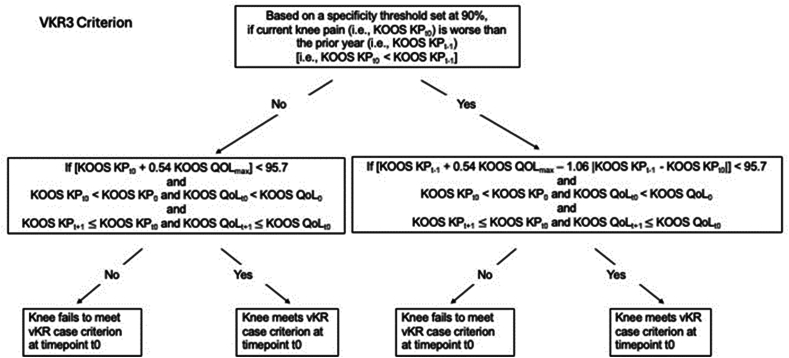
Flow chart showing how a knee with a vKR case status is identified, with the knee required to meet the vKR status criterion assuming a specificity threshold of 90 ​% (i.e., a true negative rate of 90 ​% and a false positive rate of 10 ​%). The algorithm uses data from the visit in the current year (t_0_), i.e. 48 ​M follow-up, to define if a vKR occurs and is reported as a vKR case in the following year (i.e. 60 ​M). Hence, knees with a sufficiently severe PRO status at 48 ​M, which have a high likelihood of undergoing surgical KR between 48 and 60 ​M, are defined as 60 ​M vKR case knees. The definition of vKR case is based on [top box] whether current KOOS KP (i.e., KOOS KP_t0_) is worse than (i.e., less than) the KOOS KP in the prior year (i.e., KOOS KP_t-1_) The left side of the flowchart illustrates the calculation of current KOOS knee pain being not worse than the previous year. There are three conditions: 1) If current KOOS knee pain plus 0.54 times the maximum KOOS quality of life (KOOS QoL_max_) is less than 95.7, with the maximum KOOS QOL defined as whichever is greater, the current KOOS QoL (KOOS QoL_t0_) or the KOOS QoL in the prior year (KOOS QoL_t-1_); 2) Both the current KOOS KP and the current KOOS QOL are worse than (i.e., less than) the baseline KOOS KP and KOOS QOL; and 3) Both the KOOS KP in the next year and the KOOS QOL in the next year are worse or the same as (i.e., less than or equal to) the current KOOS KP and the current KOOS QOL. If the knee meets all of these conditions, the knee is considered to have met vKR case status. The right side of the flowchart illustrates the calculation if current KOOS knee pain is worse than the previous year. Again,there are three conditions: 1) If current KOOS knee pain plus 0.54 times the maximum KOOS quality of life (KOOS QoL_max_) minus the quantity {1.06 times the absolute value of KOOS knee pain in the prior year minus current KOOS knee pain} is less than 95.7, with the maximum KOOS QOL defined as whichever is greater, the current KOOS QoL (KOOS QoL_t0_) or the KOOS QoL in the prior year (KOOS QoL_t-1_); 2) Both the current KOOS KP and the current KOOS QOL are worse than (i.e., less than) the baseline KOOS KP and KOOS QOL; and 3) Both the KOOS KP in the next year and the KOOS QOL in the next year are worse or the same as (i.e., less than or equal to) the current KOOS KP and the current KOOS QOL. If the knee meets all three of these conditions, the knee is considered to have met the vKR case criterion.

**Fig. 3:**
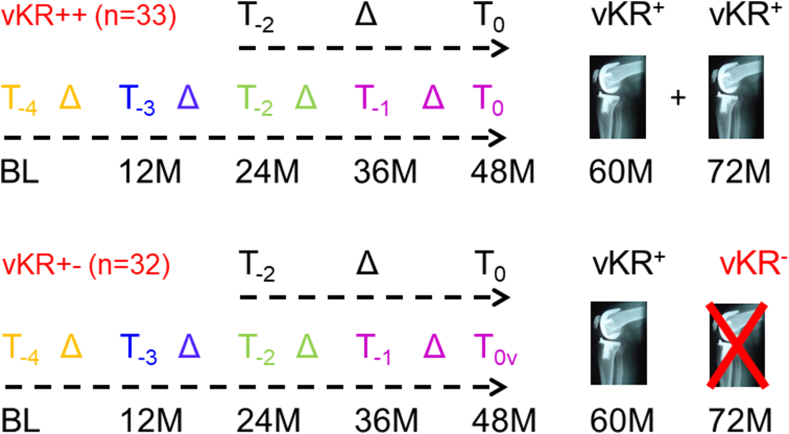
Graph showing study design and methods of the cartilage measurements: 65 OAI participants who displayed the critical symptom status above the defined threshold at 48 ​M follow-up, and thus reached vKR case (here vKR+) status at 60 ​M had quantitative cartilage analysis at up to 5 prior annual time points (T_-4_ through T_0_). Of these 33 maintained vKR status at 72 ​M (vKR++) with 31 of these having T_-2_ and T_0_ MRI data, and 32 did not maintain vKR status (vKR +/−) with 30 of these having T_-2_ and T_0_ data. One of the 61 vKR cases with MRI data did not have a control.

The definition of vKR case status in this work was derived from longitudinal clinical measures in 206 knees (187 participants – Fig. 1) undergoing KR surgery up to 60 ​M [19]. The definition was applied to 8168 knees in participants with continuous health-care coverage who did not receive a KR during this interval [19] (Fig. 1). VKR cases were selected based on the optimal logistic regression model that included Knee Injury and Osteoarthritis Outcome Score (KOOS) pain and Knee Injury and Osteoarthritis Outcome Score quality of life (QOL) scores [19]. This specific combination of PROs was derived from 18 candidate clinical variables using logistic regression (Fig. 2) [19]. The models included clinical variables observed at baseline and at t_0_ (the clinical visit before surgical KR), one-year prior to t_0_ (i.e., t_-1_), and one-year after t_0_ (i.e., t_+1_) [19]. The vKR definition is based on calculations such that a certain level of specificity can be chosen; in this case, 90 ​%, which yields a false positive rate of 10 ​% (Fig. 2). Clinical decision making of surgical KR is usually partially based on imaging (radiographic) features; however, because the intent of the current vKR definition was not to support clinical decision making, but to provide a clinically meaningful outcome measure for DMOAD trials, the vKR definition used was exclusively based on PROs, and did not incorporate information from any medical imaging modality. For our analysis, potential vKR case knees were selected from knees with levels of knee pain and/or QOL scores that placed them in the highest 10 ​% predicted probability of receiving a surgical KR. Further, vKR case knees had to have worse pain and QOL scores at 48 ​M compared with baseline, and had to be worse at 60 ​M than at 48 ​M (Fig. 2). To be able to describe the most complete 4-year trajectory of cartilage loss prior to vKR (Fig. 3), we selected knees reaching vKR case status at 60 ​M (with the patient reported outcome (PRO) symptom status defining this state being recorded at 48 ​M), but not earlier. We included knees that maintained vKR case status from 60 to 72 ​M (vKR+/+; Fig. 3) and compared their cartilage loss over 48 ​M to those who did not maintain or increase symptoms or QOL for one more other year up to 72 ​M (vKR+/−).

VKR control knees were chosen based on being in the lowest 20 ​% predicted probability of receiving a surgical KR. VKR knees in which the opposite knee reached vKR case status or received a surgical KR up to 60 ​M were not deemed eligible as controls, to improve “contrast”, given potential interactions in the perception of symptoms between both knees [28]. VKR case knees were matched 1:1 with vKR control knees by sex, age (±5years), baseline central subregions of the medial weight-bearing (central) reading visit KLG strata of 0–1, 2, 3, and 4, and the compartment with the greatest joint space narrowing grade (medial, lateral, or both). KLG and joint space narrowing were derived from central readings of fixed flexion radiographs (release 0.6) [26,27].

### 2.2 Cartilage thickness loss

Quantitative analysis of cartilage thickness was performed on sagittal double-echo steady-state MRI acquisitions [26,27] to examine the 4-year trajectory of cartilage loss (T_-4_→ T_0)_ before reaching vKR case status (Fig. 3) [7,20]. Segmentation of the femorotibial cartilages was performed at one image analysis centre (Chondrometrics GmbH, Freilassing, Germany) [20,21]. Sets of longitudinal MRI acquisitions were processed by one of 7 readers, with blinding to acquisition order and case/control status [29]. All segmentations were quality controlled by one of two experts. The mean cartilage thickness (ThCtAB.Me) was derived using Chondrometrics software [21,30]. The accuracy, reliability, test-retest precision (with repositioning), longitudinal sensitivity to change, and sensitivity to therapeutic intervention of MRI-based cartilage morphometry has been thoroughly explored and summarized in recent reviews [20,21]. Cartilage thickness of the medial and lateral compartment, of the medial and lateral tibiae, and of the weight-bearing femoral condyles was derived, as well as in 5 tibial and 3 femoral subregions [30]. Aggregate values of central medial tibial and femoral subregions were computed as central medial femorotibial (cMFTC) cartilage thickness [22], and the same was done for central lateral subregions. Reduction in cartilage thickness loss was expressed as negative value in μm. In addition to these region-specific measures, location-independent cartilage changes were studied [31,32]. For comparative reasons, the minimal medial joint space width (mJSW) loss was determined from fixed flexion radiographs for vKR case and control knees between T_-2_ and T_0_ [8], since mJSW has been traditionally accepted by regulatory agencies.

### 2.3 Statistical analysis

All tests were performed using SAS software (version 9.4, SAS Institute, Cary, NC).

As in an initial study in surgical KRs [6], change in cMFTC cartilage thickness was used as the primary analytic focus and total medial compartment (MFTC) cartilage thickness as the secondary. Other regions were regarded exploratory. This choice was made because the cMFTC represents the central weight-bearing area of the MFTC and was observed to be the region with the greatest sensitivity (highest standardized response mean) of cartilage loss in OAI knees [33,34], and because no previous analysis provided guidance as to which femorotibial subregion may discriminate better between vKR case and control knees. The T_-2_→T_0_ observation interval was used in the primary analysis, since previous work showed this interval to best discriminate between surgical KR and non-replaced controls [7], likely due to the greater robustness and more favorable ratio of the signal and the measurement error compared with one-year observation intervals. The other observation intervals, describing the entire 4-year trajectory of cartilage loss by MRI in vKR case and matched vKR control knees, were run as descriptive analyses.

Statistical comparisons between vKR case and vKR control knees included paired t-tests and case-control conditional logistic regression odd ratios (OR) per standard deviation. Correlations between knees of 3 participants with bilateral vKR case status and for one person contributing two vKR control knees were accounted for by the robust sandwich estimator for the covariance matrix of the regression coefficients [35,36]. Robustness of the comparisons was assessed by performing additional adjustment for the effects of standard categories of the body mass index (BMI: normal/overweight/obese) at the beginning of each observation interval. Sensitivity analyses were performed to compare knees that maintained vKR status at 72 ​M (vKR+/+) vs. those that did not (vKR+/−). Non-parametric Kruskal Wallis tests were used, given that the distribution of some of the variables was skewed (Fig. 3).

## 3 Results

### 3.1 Study sample

117 knees of 107 OAI participants reached vKR case status for the first time at 60 ​M, and these, 65 knees (of 62 OAI participants) a) had longitudinal MRIs spanning the full observation period prior to reaching 60 ​M vKR case status and b) could be successfully matched to a vKR control with low probability of becoming a surgical KR. This was done using the demographic, clinical and radiographic data shown in Table 1.

**Table 1:** Baseline demographic and radiographic variables of knees with vKR case vs. vKR control status (mean ​± ​standard deviation).

		vKR case (n ​= ​65)	vKR control (n ​= ​65)	p value (case vs. control)^a^
Age (y)		62.8 ​± ​9.3	62.6 ​± ​9.3	0.87
Sex (No women)		34	34	
BMI (kg/m^2^)		29.3 ​± ​4.5	28.5 ​± ​5.0	0.31
WOMAC pain score		2.91 ​± ​2.86	3.28 ​± ​3.09	0.48
KOOS pain score		81.2 ​± ​14.0	78.9 ​± ​15.6	0.38
KOOS QOL score		62.2 ​± ​17.0	66.0 ​± ​18.5	0.23
KL	Grade			0.59
No (%)	0	17 (26)	12 (18)	
	1	4 (6)	9 (14)	
	2	17 (26)	17 (26)	
	3	18 (28)	18 (28)	
	4	9 (14)	9 (14)	
Medial mJSW (mm)		4.04 ​± ​1.93	3.67 ​± ​1.53	0.30

a Group difference tests by Chi Squared, Fisher's Exact Test, and *t*-test; y ​= ​years, No ​= ​number, m ​= ​male; BMI ​= ​body mass index; WOMAC = Western Ontario and McMaster Universities Arthritis Index; KOOS = Knee Injury and Osteoarthritis Outcome Score; QOL ​= ​quality of life; KL = Kellgren Lawrence; mJSW ​= ​minimal radiographic joint space width obtained from fixed flexion radiographs.

Of the 65 knees pairs with longitudinal MRIs that could be successfully matched the baseline KLG status (i.e., 5 years prior to reaching vKR case status) is shown in Table 1. The 52 vKR case knees, for which no vKR control match could be found did not differ noticeably from the 65 successfully matched in terms of demographic data; however, they displayed a slightly different KL distribution, with 6 (11.5 ​%) KLG0, 6 (11.5 ​%) KLG1, 24 (46 ​%) KLG2, 15 (29 ​%) KLG3, and 1 (2 ​%) KLG4). Of the 65 vKR case knees, 33 maintained pain and QOL levels up to 72 ​M, whereas 32 did not.

### 3.2 Medial compartment cartilage loss during T_-2_→T_0_

60 of the 65 case-control pairs had MRI at T_-2_→T_0,_ and 27 had a mJSW measurement. In the primary statistical comparison, vKR case knees displayed significantly greater cMFTC cartilage loss than the vKR control knees (−151 ​± ​337 vs. −38 ​± ​249 ​μm; p ​= ​0.005 [paired-t]; OR 1.95 (95 ​% confidence interval [CI]: 1.23, 3.08; Table 2). Findings persisted after adjustment for BMI at T_-2_ (aOR ​= ​1.99). In the secondary statistical comparison, total MFTC cartilage loss also was significantly greater in vKR case than vKR control knees (−101 ​± ​207 vs. −26 ​± ​150 ​μm; p ​= ​0.007 [paired-t]; OR 1.75 (95% confidence interval [CI]: 1.18, 2.59; aOR 1.76, Table 2). There were slightly greater differences in central weight-bearing femoral (ccMF) cartilage loss between vKR case and vKR control knees than in central medial tibia cartilage thickness loss (Table 2). Medial mJSW change (T_-2_→T_0_) also was greater in vKR case knees (−458 ​± ​663 vs. −278 ​± ​743 ​μm; OR 1.28 (95 ​% CI 0.80, 2.06), but the difference did not reach statistical significance (p ​= ​0.35; paired *t*-test). For comparison, the cMFTC cartilage loss was −167 ​± ​332 vs. −33 ​± ​246 ​μm in those 27 vKR case vs. VKR control knees with mJSW measurement, with an OR of 1.81 (95 ​% CI ​= ​0.94; 3.5; paired *t*-test p value ​= ​0.08).

**Table 2:** Longitudinal 2-year cartilage thickness change (in μm) prior to reaching virtual knee replacement (vKR) status for vTR ​+ ​cases and matched vTR-controls (T_-2_ → T_0_) for different variables (n ​= ​60/60^a^).

Variable	vKR cases		vKR controls		Paired-t	Non-adjusted log. regress.		Adjusted log. regress	
	Mean	SD	Mean	SD	p-value	OR (95 ​% CI)	p-value	OR (95 ​% CI)	p-value
cMFTC	−151	337	−38	249	0.005	1.95 (1.23.3.08)	0.004	1.99 (1.25.3.18)	0.004
MFTC	−101	207	−26	150	0.007	1.75 (1.18.2.59)	0.005	1.76 (1.15.2.68)	0.009
cLFTC	−101	274	−29	229	0.067	1.48 (0.92.2.39)	0.107	1.43 (0.88.2.32)	0.145
LFTC	−50	180	−24	156	0.290	1.27 (0.81.1.98)	0.301	1.24 (0.79.1.95)	0.354
cMT	−71	164	−27	163	0.089	1.46 (0.96.2.22)	0.079	1.47 (0.92.2.37)	0.108
ccMF	−80	235	−11	127	0.016	1.51 (1.09.2.09)	0.013	1.57 (1.12.2.18)	0.008
Thinning score	−1149	851	−851	682	0.011	1.76 (1.05.2.96)	0.032	1.69 (1.00.2.86)	0.049
Thicking score	532	434	635	440	0.165	0.75 (0.49.1.15)	0.190	0.75 (0.49.1.15)	0.182

a Four case knees and one control knees did not have a readable image at either T0 or T-2. Log. Regress ​= ​logistic regression model; OR ​= ​odds ratio; 95 ​% CI ​= ​95 ​% confidence interval; SD ​= ​standard deviation; cMFTC ​= ​central subregions of the medial femorotibial compartment (cMT ​+ ​ccMF); MFTC ​= ​total medial femorotibial compartment; cLFTC ​= ​central subregions of the lateral femorotibial compartment; LFTC ​= ​total lateral femorotibial compartment; cMT ​= ​central subregion of the medial tibia; ccMF ​= ​central subregions of the medial weight-bearing (central) medial femur; Thinn score ​= ​location-independent subregion thinning score; Thick score ​= ​location-independent subregion thickening score.

### 3.3 Other measures of cartilage loss and other observation intervals

T_-2_→T_0_ cartilage loss in the central and total lateral compartments also appeared greater in vKR case than in vKR control knees, but the difference failed to reach statistical significance (Table 2). The location-independent measure of cartilage loss (total subregion cartilage thinning score) differed significantly between vKR case and vKR control knees during T_-2_→T_0,_ with an OR (1.76) similar to that of total MFTC (1.75); the total subregion cartilage thickening score, in contrast, did not differ between vKR case and vKR control knees.

Analysis of medial compartment cartilage loss during annual observation intervals revealed significant differences between vKR case and vKR control knees during T_-1_→T_0_, but was less during the preceding annual observation intervals (Table 3; Fig. 3). Yet, it is noteworthy that in vKR case knees the mean cartilage loss during the T_-4_→T_-3_ intervals was still more than twice that in vKR controls (Table 3; Fig. 3).

**Table 3:** Longitudinal cartilage thickness change (in μm) in the central medial femorotibial compartment for various annual intervals prior to reaching virtual knee replacement (vKR) status for vTR cases and matched controls.

Inteval n ​=		vKR cases		vKR controls		Paired-t	Non-adjusted log. regress.		Adjusted log.regress	
		Mean	SD	Mean	SD	p-value	OR (95 ​% CI)	p-value	OR (95 ​% CI)	p-value
T_-1_→T_0_	60	−30	278	76	228	0.02	1.67 (1.11, 2.50)	0.01	1.63 (1.12, 2.36)	0.01
T_-2_→T_-1_	59	−125	301	−94	240	0.51	1.13 (0.78, 1.63)	0.52	1.12 (0.77, 1.64)	0.55
T_-3_→T_-2_	59	−96	307	−62	197	0.48	1.11 (0.86, 1.43)	0.43	1.10 (0.85, 1.42)	0.49
T_-4_→T_-3_	60	−65	233	−30	172	0.34	1.17 (0.86, 1.59)	0.31	1.15 (0.85, 1.56)	0.38

Log. Regress ​= ​logistic regression model; OR ​= ​odds ratio; 95 ​% CI ​= ​95 ​% confidence interval; SD ​= ​standard deviation; T_-1_→T_0_ ​= ​annual observation interval in the year prior to reaching vKR status (36 month follow-up [M] →48 ​M); T_-2_→T_-1_ ​= ​annual observation interval 2 years prior to reaching vKR status (24 ​M→36 ​M); T_-3_→T_-2_ ​= ​annual observation interval 3 years prior to reaching vKR status (12 ​M→24 ​M); T_-4_→T_-3_ ​= ​annual observation interval 3 years prior to reaching vKR status (BL [baseline]→12 ​M).

Cartilage loss in either the medial or lateral compartment did not differ between knees who maintained vKR case status at 72 ​M (vKR+/+) vs. those who did not (vKR+/−), with p-values between 0.40 and 0.97 (Table 4).

**Table 4:** Longitudinal 2-year cartilage thickness change (in μm) prior to reaching virtual knee replacement (vKR) status, for vKR cases who maintained case status at 72 month follow-up (vKR++, n ​= ​31) versus those who did not (vKR+/−; n ​= ​30).

Variable	vKR++		vKR +/−		Kruskall Wallis
	Mean	SD	Mean	SD	p-value
cMFTC	−150	374	−157	295	0.73
MFTC	−105	228	−99	183	0.97
cLFTC	−81	286	−129	261	0.40
LFTC	−61	210	−43	144	0.90
cMT	−67	189	−75	133	0.48
ccMF	−83	221	−82	251	0.57

cMFTC ​= ​central subregions of the medial femorotibial compartment (cMT ​+ ​ccMF); MFTC ​= ​total medial femorotibial compartment; cLFTC ​= ​central subregions of the lateral femorotibial compartment; LFTC ​= ​total lateral femorotibial compartment; cMT ​= ​central subregion of the medial tibia; ccMF ​= ​central subregions of the medial weight-bearing (central) medial femur; note that the reason for 61 vKR ​+ ​case knees being included in this analysis and only 60 in Table 1 was that one vKR ​+ ​case knee did not have a matched vTKR control.

## 4 Discussion

This is the first study to explore quantitative MRI cartilage thickness loss in knees prior to reaching vKR case vs. control status. Central medial femorotibial compartment cartilage loss in vKR case knees significantly exceeded that in vKR control knees over the two years prior to reaching that symptom status, and similar observations were made over the four-year trajectory. The rate of medial cartilage loss did not differ between vKR case knees that maintained that status for another year (vKR+/+) vs. case knees who did not (vKR+/−).

117 knees fulfilled vKR case status for the first time at the time point studied (60 ​M). This is a small percentage of OAI knees, but more than the surgical KRs observed at 60 ​M (n ​= ​67). A limitation of our study is that vKR case knees were chosen after real KRs were eliminated and that, in this first step, no attempt was made to provide a composite or multi-component endpoint of combined vKR and surgical KR analysis. Although the relatively small vKR case and control samples represent a limitation of this study, very large cohorts will be required to identify larger matched vKR case/control samples. Although there was a reasonable rationale of excluding participants without insurance coverage, because the vKR definition used here was developed based on surgical KR, future “application-orientated” studies, may include patients without such coverage. Controls here were chosen to have a low probability of being vKR. Investigations that regard vKR as a purely dichotomous multi component outcome, where controls would be all knees not representing a vKR, and knees contralateral to KR or vKR knees are also included, will need to be supported by larger samples and will be associated with higher cost than this initial study. The T^−1^ to T^0^ cartilage thickness changes appeared smaller than in prior intervals, and positive in the controls. A potential explanation is that the 36 ​M cartilage data were processed at a later time point, albeit by the same readers with reference to the other (segmented) images, but with full blinding to vKR case/control status. Also, the current cartilage analysis was focused on femorotibial cartilage loss, as are most current epidemiological and interventional studies, whereas pathology of the femoropatellar joint may have also made a relevant contribution to the pain and QOL levels that led to vKR case status. Further, vKR case status may have been impacted by trauma or degenerative anterior cruciate ligament tears [37], and structural abnormalities related to cartilage loss (e.g., the hip knee angle [38], meniscal tears [39], or other structural joint pathologies [40,41]), which were not included in the matching or statistical adjustment process. A strength of the approach, however, is that each vKR case was closely matched 1:1 to a vKR control, the latter with a very low probability of becoming a vKR. Further, up to 5 annual MRI acquisitions were analyzed in each vKR case/control pair, providing a 4-year trajectory of cartilage loss in knees that reached vKR case status at 60 ​M. A limitation to mention in this context: the OAI provided data up to 96 ​M, but not annual MRIs, so 72 ​M and 96 ​M MRI data were not included in the current analysis.

The biannual cartilage loss in the total MFTC prior to reaching vKR case status (−101 ​± ​207 ​μm) was lower than that previously observed in surgical KRs (−254 ​± ​414 ​μm) [7], but it was significantly greater than in vKR control knees. The medial compartment difference of the biannual cartilage loss between vKR case and control knees was 75 ​μm, which is noticeably more than the 50 ​μm difference (for total joint cartilage thickness change) between the placebo-vs. the sprifermin-treated arm (100 ​μm given every 6 months) in the FORWARD study, a recent DMOAD RCT [23]. This between-group difference in cartilage thickness loss (50 ​μm) was shown to translate into a statistically significant and clinically relevant benefit (greater than the minimally clinically important difference ​= ​MCID) at 36 ​M [23] and at 60 ​M [25] in a post-hoc study of the so-called "subcohort at risk". It is important to note that the significant discrimination between vKR case and control knees was observed even after surgical KRs had been eliminated from the study, so the discrimination applied to knees that did not actually receive surgical KR in medical practice.

The sample studied here included knees in which PROs suggested that surgical replacement was indicated, but these were not actually replaced, potentially due to patient preference, surgeon choice (radiographic status not yet suitable), medical, or socioeconomic reasons. The discrimination between vKR case and control knees in the current study (non-adjusted OR ​= ​1.75 for total MFTC cartilage loss) parallels, in a relative sense, that between surgical KR cases vs. non-replaced controls (non-adjusted OR for total MFTC cartilage loss ​= ​1.40) in an earlier study [7]. The effect sizes are not directly comparable, because ORs were expressed as SDs of different sets of controls. Additionally, the effect size for the current vKR case analysis was driven by low and more uniform rates of cartilage loss in vKR controls. In vKR case and control pairs who had radiographic mJSW measurement, the observed difference in cMFTC cartilage loss was greater than that in mJSW loss. The study was not designed nor powered to identify a significant difference in the performance of MRI vs. radiography, however [8].

As was seen in prior surgical KR vs. non-KR cartilage thickness analysis, the discrimination between vKR case and control knees was greater when relying on medial rather than on lateral femorotibial cartilage loss, although differences in cartilage loss in the central lateral femorotibial compartment almost reached statistical significance. Location-independent analysis provided similar differentiation of vKR case vs. control knees as did the MFTC cartilage loss.

The current results are to some extent in contrast to those reported by the Foundation for the National Institutes of Health (fNIH) OA Biomarkers Consortium [42,43]. Whereas there, quantitative change in MRI cartilage loss was significantly associated with radiographic but not with (isolated) symptomatic progression of knee OA [42,43], we identify a clear relationship of cartilage loss with prospective symptom worsening in the current study. Potential reasons for the apparent discrepancy include a) the use of WOMAC pain as a PRO in the fNIH Consortium, whereas a more complex criterion (KOOS pain and QOL scores at different time points) was used here, b) that radiographic progression was actively ruled out in the fNIH subsample with isolated symptomatic progression in the fNIH, whereas vKR cases in our current analysis were not precluded from displaying radiographic change, and c) that controls in the current study were specifically selected to have a low risk of undergoing surgical KR, whereas in the fNIH study, any participant not meeting the case definition could serve as a control.”.

In prior surgical KR analyses, the difference in cartilage loss was most apparent during the two years prior to reaching vKR case status [7], which is in line with the observation of a relatively steep increase in pain and reduction in QOL prior to KR surgery in the OAI [19]. These findings stress that fast structural progression, defined by high rates of cartilage loss, is related not only to surgical KR as a “hard” clinical outcome, but also to an objectively defined worsening of PROs, here defined as vKR case status. This provides important evidence for the clinical usefulness and validity of quantitative cartilage measurement by MRI as an imaging biomarker, to be deployed in clinical DMOAD trials. It does not mean cartilage is the only or even the most relevant tissue in the association of structural pathology with clinical outcomes, as other tissues are also associated with subsequent surgical KR [11,[44], [45], [46]]. Yet, structural pathology in these tissues is also known to be significantly associated with quantitative cartilage loss [40,41], the latter representing a quantitative measure of disease progression that decreases more or less continuously but, unlike other tissues, does not fluctuate. Fully automated measurement technology [47,48] will permit to scale this measure to larger samples The current data provide an important step in further qualifying quantitative cartilage loss as an imaging biomarker in OA [3].

An OARSI/OMERACT initiative previously attempted to establish criteria for being considered a candidate for joint replacement in the hip or knee. The authors reported a relatively low sensitivity and concluded that there was too much overlap in symptoms between KR cases and controls to be able to establish a reasonable threshold [49,50]. Although there is also overlap in our vKR PRO status, the current results are encouraging, as they suggest that the definition used here for determining vKR case status is similarly related to differences in structural progression (i.e. cartilage loss) as is surgical KR [6,7]. It also may represent an efficient endpoint for qualifying “cartilage thickness change” as a potential surrogate endpoint to increase the feasibility of performing more short-term clinical trials (1–2 years) to establish a proof-of-concept (POC). A qualified imaging endpoint may also be pivotal in obtaining “accelerated approval” for a DMOAD in a larger phase-2 trial, with post-approval verification based on clinical outcomes (e.g. vKR) [3].

In conclusion, this study demonstrates that knees reaching a vKR case status defined by clinical PROs experienced substantially and statistically significantly greater cartilage loss than vKR controls with a low probability of becoming a surgical KR case. This appears independent of whether fulfilling this status is maintained for another year after reaching vKR case status and suggests that the vKR definition developed is useful, and that cartilage loss and worsening PROs (pain and function) are associated with each other. These results extend previous work that validated MRI assessed cartilage thickness as an imaging biomarker for structure modification intervention trials.

## Author contributions

All authors have made substantial contributions to:

(1) The conception and design of the study, or acquisition of data, or analysis and interpretation of data,

(2) Drafting the article or revising it critically for important intellectual content,

(3) Final approval of the version to be submitted.

## Ethical approval information

The OAI study and public use of its clinical and imaging data was approved by the Committee on Human Research, that is the Institutional Review Board for the University of California, San Francisco (UCSF), and its affiliates (Approval No 10–00532). https://oai.epi-ucsf.org/datarelease/docs/IRBApproval.pdf. UCSF holds Office of Human Research Protections Federalwide Assurance number FWA00000068. The participants gave informed consent before taking part in the study.

## Role of the funding source

The study and image acquisition was supported by the OAI, a public-private partnership comprised of five contracts (N01-AR-2-2258; N01-AR-2-2259; N01-AR-2-2260; N01-AR-2-2261; N01-AR-2-2262) funded by the National Institutes of Health, a branch of the Department of Health and Human Services, and conducted by the OAI Study Investigators. Private funding partners include Pfizer, Inc.; Novartis Pharmaceuticals Corporation; Merck Research Laboratories; and GlaxoSmithKline. Private sector funding for the OAI is managed by the Foundation for the National Institutes of Health.

The image analysis of this study was partly funded by MerckKGaA (Darmstadt, Germany), by a contract with the University of Pittsburgh (Pivotal OAI MRI Analyses POMA: NIH/NHLBI Contract No. HHSN2682010000 21C), by a vendor contract from the OAI coordinating center at University of California, San Francisco (N01-AR-2-2258), and by an ancillary grant to the OAI held by Northwestern University (NIH/NIAMS R01 AR052918 [Sharma]).

The statistical data analysis was funded by a contract with the University of Pittsburgh (Pivotal OAI MRI Analyses POMA: NIH/NHLBI Contract No. HHSN2682010000 21C) and the University of Pittsburgh Multidisciplinary Clinical Research Center for Rheumatic and Musculoskeletal Diseases (P60 AR054731).

The sponsors were not directly involved in the design and conduct of the study; collection, management, analysis, and interpretation of the data; and preparation, review, or approval of the manuscript. However, the co-authors participating in the study (and partly being affiliated with study sponsors) were involved in all above aspects of the study. The statistical analysis of the data (based on the entire raw data set and evaluation of the study protocol, and pre-specified plan for data analysis) was conducted by an independent statistical team at an academic institution (the University of Pittsburgh) which was independent of the commercial sponsors. No compensation or funding was received from a commercial sponsor for conducting the statistical analyses.

The statistical analysis and writing of this article were independent from and not contingent upon approval from the study sponsors.

## Declaration of competing interest

Felix Eckstein is co-owner and CEO of Chondrometrics GmbH, a company providing MR image analysis services to academic researchers and to the pharmaceutical industry. He has provided consulting services to MerckSerono, Galapagos/Servier, Kolon Tissue Gene, Novartis, Peptinov, Formation Bio, 4P Pharma, Sanofi, and Artialis.

Wolfgang Wirth has a part time employment with Chondrometrics GmbH and is a co-owner of Chondrometrics GmbH.

Ali Guermazi is President and co-owner of the Boston Core Imaging Lab (BICL). He has provided consulting services to TissueGene, Novartis, ICM, Paradigm, Formation Bio, 4Moving, Scarcell Therapeutics, Pacira, Coval, Medipost, Levicept and Peptinov. He is a Co-Editor-in-Chief of *Skeletal Radiology*.

Frank Roemer is shareholder and CMO of Boston Core Imaging Lab (BICL), LLC. He received an institutional grant from the Else Kröner-Fresenius-Stiftung. He has provided consulting services to Grünenthal. He is Editor-in-Chief of the journal *Osteoarthritis Imaging*.

Christoph Ladel was an employee of MerckKGaA and has provided consulting services to ReumaNederland, Charité, Formation Bio, Gordian Bio, Integra Holdings, CurNova and Regenosine. Travel support was received by netwOArk (COST action).

C. Kent Kwoh Manuscript funding: NIH; Grants or contracts: AbbVie, Artiva, Lilly, BMS, Cumberland, Pfizer, GSK, Galapagos; Consulting fees: Trial Spark, Express Scripts, GSK, TLC Biosciences, AposHealth; Payment or honoraria for lectures: Focus Medical Communications, Participation on Data Safety; Monitoring or Advisory Board: Moebius Sun, Novartis, Xalud, Kolon Tissue Gene; Leadership or fiduciary role on a board: International Chinese Osteoarthritis Research Society (ICOARS).

DJH is the Editor of the osteoarthritis section for UpToDate and co-Editor-in-Chief of *Osteoarthritis and Cartilage*. He provides consulting advice on scientific advisory boards for Haleon, TLCBio, Novartis, TissueGene, Sanofi, and Enlivex.

Neither Michael Nevitt nor Leena Sharma have conflicts of interest to declare.
